# Supplementary material for: Wastewater-Based Epidemiology for Viral Surveillance from an Endemic Perspective: Evidence and Challenges
Source: Viruses. 2024 Mar 20;16(3):482. doi: 10.3390/v16030482 (PMC10975420; doi:10.3390/v16030482)
Supplement: Supplementary file 1 [file viruses-16-00482-s001.zip › viruses-2856891-supplementary.pdf]

## Supplementary Materials

# Wastewater-Based Epidemiology for Viral Surveillance from an Endemic Perspective: Evidence and Challenges

**Marco Verani †, Alessandra Pagani †, Ileana Federigi *, Giulia Lauretani, Nebiyu Tariku Atomsa, Virginia Rossi, Luca Viviani and Annalaura Carducci**

Laboratory of Hygiene and Environmental Virology, Department of Biology, University of Pisa, Via S. Zeno 35/39, 56123 Pisa, Italy; marco.verani@unipi.it (M.V.); alessandra.pagani@phd.unipi.it (A.P.); ileana.federigi@unipi.it (I.F.); g.lauretani@studenti.unipi.it (G.L.); nebiyu.atomsa@phd.unipi.it (N.T.A.); v.rossi33@studenti.unipi.it (V.R.); luca.viviani97@gmail.com (L.V.); annalaura.carducci@unipi.it (A.C.)

* Correspondence: ileana.federigi@unipi.it; Tel.: +39-0502213646

† These authors contributed equally to this work.

**Table S1**. Occurrence and viral load of HAdV according to WWTPs and seasons.

| Season | WWTP type | Positive samples (n°, %) | Viral load Log $_{10}$(GC/100,000 inh/day) |
|---|---|---|---|
| **Autumn 2021** | WWTP1 | 9/12, 75.0% | 8.8 ± 2.7 |
| | WWTP2 | 9/12, 75.0% | 8.6 ± 2.6 |
| | WWTP3 | 9/13, 69.2% | 8.3 ± 2.8 |
| | WWTP4 | 7/12, 58.3% | **7.7 ± 2.9** |
| | **Total of WWTPs** | **34/49, 69.4%** | **8.4 ± 2.7** |
| **Winter 2022** | WWTP1 | 8/10, 80.0% | 9.4 ± 2.7 |
| | WWTP2 | 10/10, 100% | 10.5 ± 0.5 |
| | WWTP3 | 5/12, 41.7% | 6.8 ± 3.2 |
| | WWTP4 | 3/12, 33.3% | 5.8 ± 2.7 |
| | **Total of WWTPs** | **26/44, 19.1%** | **8.0 ± 3.1** |
| **Spring 2022** | WWTP1 | 12/13, 92.3% | 10.9 ± 2.0 |
| | WWTP2 | 13/13, 100% | 11.2 ± 0.4 |
| | WWTP3 | 10/13, 76.9% | 8.8 ± 2.7 |
| | WWTP4 | 12/13, 92.3% | 9.9 ± 1.7 |
| | **Total of WWTPs** | **47/52, 90.4%** | **9.5 ± 2.7** |
| **Summer 2022** | WWTP1 | 13/13, 100% | 11.7 ± 0.4 |
| | WWTP2 | 13/13, 100% | 10.9 ± 0.3 |
| | WWTP3 | 12/13, 92.3% | 9.8 ± 1.8 |
| | WWTP4 | 13/13, 100% | 10.7 ± 0.6 |
| | **Total of WWTPs** | **51/52, 98.0%** | **10.8 ± 1.2** |
| **One year** | WWTP1 | 42/48, 87.5% | 10.3 ± 2.3 |
| | WWTP2 | 45/48, 93.7% | 10.3 ± 1.6 |
| | WWTP3 | 36/51, 78.6% | 8.5± 2.8 |
| | WWTP4 | 35/50, 70.0% | 8.6 ± 2.9 |
| | **Total of WWTPs** | **158/197, 80.2%** | **9.4 ± 2.6** |

**Table S2**. Occurrence and viral load of enterovirus (EV) according to WWTPs and seasons.

| Season | WWTP type | Positive samples (n°, %) | Viral load Log $_{10}$(GC/100,000 inh/day) |
|---|---|---|---|
| **Autumn** | WWTP1 | 6/12, 50.0% | 6.5 ± 2.5 |
| | WWTP2 | 0/12, 0% | 4.0 ± 0.1 |
| | WWTP3 | 2/13, 15.4% | 4.7 ± 1.6 |
| | WWTP4 | 7/12, 58.3% | 6.4 ± 2.3 |
| | **Total of WWTPs** | **15/49, 30.6%** | **5.4 ± 2.1** |
| **Winter** | WWTP1 | 3/10, 30.0% | 5.5 ± 2.2 |
| | WWTP2 | 0/10, 0% | 4.0 ± 0.0 |
| | WWTP3 | 1/12, 8.3% | 4.3 ± 1.4 |
| | WWTP4 | 2/12, 16.7% | 3.9 ± 0.0 |
| | **Total of WWTPs** | **6/44, 13.6%** | **4.6 ± 1.6** |
| **Spring** | WWTP1 | 9/13, 69.2% | 7.5 ± 2.4 |
| | WWTP2 | 2/13, 15.4% | 4.5 ± 1.4 |
| | WWTP3 | 10/13, 76.9% | 7.1 ± 1.9 |
| | WWTP4 | 10/13, 76.9% | 7.6 ± 2.1 |
| | **Total of WWTPs** | **31/52, 59.6%** | **6.7 ± 2.3** |
| **Summer** | WWTP1 | 12/13, 92.3% | 8.0 ± 1.4 |
| | WWTP2 | 2/13, 15.4% | 4.5 ± 1.3 |
| | WWTP3 | 11/13, 84.6% | 7.4 ± 1.7 |
| | WWTP4 | 10/13, 76.9% | 7.2 ± 1.9 |
| | **Total of WWTPs** | **35/52, 67.3%** | **6.8 ± 2.1** |
| **One year** | WWTP1 | 30/48, 62.5% | 7.0 ± 2.3 |
| | WWTP2 | 4/48, 8.3% | 4.3 ± 1 |
| | WWTP3 | 24/51, 47% | 5.9 ± 2.1 |
| | WWTP4 | 29/50, 58.0% | 6.6 ± 2.2 |
| | **Total of WWTPs** | **87/197, 44.2%** | **5.9 ± 2.2** |

**Table S3**. Occurrence and viral load of norovirus genogroup II (NoVggII) according to WWTPs and seasons.

| Season | WWTP type | Positive samples (n°, %) | Viral load Log $_{10}$(GC/100,000 inh/day) |
|---|---|---|---|
| **Autumn** | WWTP1 | 11/12, 91.7% | 9.1 ± 1.6 |
| | WWTP2 | 8/12, 66.7% | 7.5 ± 2.6 |
| | WWTP3 | 13/13, 100% | 8.4 ± 2.2 |
| | WWTP4 | 12/12, 100% | 9.5 ± 0.4 |
| | **Total of WWTPs** | **44/49, 89.8%** | **8.6 ± 2.0** |
| **Winter** | WWTP1 | 10/10, 100% | 9.7 ± 0.6 |
| | WWTP2 | 8/10, 80.0% | 8.0 ± 2.2 |
| | WWTP3 | 9/12, 75.0% | 7.4 ± 2.5 |
| | WWTP4 | 11/12, 91.7% | 9.2 ± 1.9 |
| | **Total of WWTPs** | **38/44, 86.4%** | **8.7 ± 2.0** |
| **Spring** | WWTP1 | 11/13, 84.6% | 8.6 ± 2.0 |
| | WWTP2 | 9/13, 69.2% | 7.5 ± 2.5 |
| | WWTP3 | 11/13, 84.6% | 8.1 ± 2.0 |
| | WWTP4 | 11/13, 84.6% | 8.4 ± 2.0 |
| | **Total of WWTPs** | **42/52, 80.8%** | **8.2 ± 2.1** |
| **Summer** | WWTP1 | 12/13, 92.3% | 9.2 ± 1.7 |
| | WWTP2 | 8/13, 61.5% | 6.7 ± 2.3 |
| | WWTP3 | 11/13, 84.6% | 8.6 ± 2.2 |
| | WWTP4 | 13/13, 100% | 9.1 ± 0.7 |
| | **Total of WWTPs** | **44/52, 84.6%** | **8.4 ± 2.1** |
| **One year** | WWTP1 | 44/48, 91.7% | 9.1 ± 1.6 |
| | WWTP2 | 33/48, 68.7% | 7.4 ± 2.4 |
| | WWTP3 | 42/51, 82.3% | 8.2 ± 2.1 |
| | WWTP4 | 47/50, 94% | 9.1 ± 1.4 |
| | **Total of WWTPs** | **166/197, 84.3%** | **8.5 ± 2.0** |

**Table S4.** Occurrence and viral load of SARS-CoV-2 according to WWTPs and seasons.

| Season | WWTP type | Positive samples (n°, %) | Viral load Log $_{10}$(GC/100,000 inh/day) |
|---|---|---|---|
| **Autumn** | WWTP1 | 6/12, 50.0% | 6.4 ± 1.9 |
| | WWTP2 | 5/12, 41.7% | 6.0 ± 1.8 |
| | WWTP3 | 8/13, 61.5% | 6.8 ± 1.8 |
| | WWTP4 | 9/12, 75.0% | 7.2 ± 1.8 |
| | Total of WWTPs | 28/49, 57.1% | 6.6 ± 1.8 |
| **Winter** | WWTP1 | 8/10, 80.0% | 8.1 ± 1.9 |
| | WWTP2 | 4/10, 40.0% | 6.0 ± 2.1 |
| | WWTP3 | 5/12, 41.7% | 6.5 ± 2.3 |
| | WWTP4 | 7/12, 58.3% | 6.6 ± 2.3 |
| | **Total of WWTPs** | **24/44, 54.5%** | **6.8 ± 2.2** |
| **Spring** | WWTP1 | 10/13, 76.9% | 7.8 ± 1.9 |
| | WWTP2 | 7/13, 53.8% | 6.6 ± 2.1 |
| | WWTP3 | 5/13, 38.5% | 6.1 ± 2.3 |
| | WWTP4 | 6/13, 46.1% | 6.3 ± 2.1 |
| | **Total of WWTPs** | **28/52, 53.8%** | **6.7 ± 2.1** |
| **Summer** | WWTP1 | 6/13, 46.1% | 6.5 ± 2.3 |
| | WWTP2 | 5/13, 38.5% | 5.9 ± 1.9 |
| | WWTP3 | 4/13, 30.8% | 5.7 ± 2.2 |
| | WWTP4 | 5/13, 38.5% | 6.1 ± 2.1 |
| | **Total of WWTPs** | **20/52, 38.5%** | **6.0 ± 2.1** |
| **One year** | WWTP1 | 30/48, 62.5% | 7.2 ± 2.1 |
| | WWTP2 | 21/48, 43.7% | 6.1 ± 1.9 |
| | WWTP3 | 22/51, 43.1% | 6.2 ± 2.1 |
| | WWTP4 | 27/50, 54% | 6.6 ± 2.1 |
| | **Total of WWTPs** | **100/197, 50.8%** | **6.5 ± 2.1** |
